# Attitudes and experiences with fentanyl contamination of methamphetamine: exploring self-reports and urine toxicology among persons who use methamphetamine and other drugs

**DOI:** 10.1186/s12954-023-00782-1

**Published:** 2023-04-20

**Authors:** Raminta Daniulaityte, Lance Ruhter, Matthew Juhascik, Sydney Silverstein

**Affiliations:** 1 College of Health Solutions, Arizona State University, 425 N 5Th Street, ABC 121, Phoenix, AZ 85004 USA; 2 Montgomery County Coroner’s Office and Crime Laboratory, Dayton, OH USA; 3 Department of Population and Public Health Sciences, Center for Interventions, Treatment, and Addictions Research, Boonshoft School of Medicine, Wright State University, Dayton, OH USA

**Keywords:** Methamphetamine, Fentanyl, Fentanyl analogs, Xylazine, Drug contamination, Toxicology, Harm reduction, Polydrug use

## Abstract

**Background:**

There are growing concerns about illicitly manufactured fentanyl (IMF) contamination of methamphetamine. This study aims to characterize the lay views and experiences with IMF-contaminated methamphetamine (IMF/meth) and identify participants with unknown IMF exposures through urine toxicology analysis.

**Methods:**

Between December-2019 and November-2021, structured interviews were conducted with 91 individuals who reported past 30-day use of methamphetamine and resided in Dayton, Ohio, USA. Lab-based urine toxicology analyses were conducted to identify fentanyl/analogs, methamphetamine, and other drugs. Bivariate analyses were conducted to identify characteristics associated with attitudes and experiences with IMF/meth, and unknown IMF exposures.

**Results:**

The majority (95.6%) of the study participants were non-Hispanic white, and 52.7% were female. Past 30-day use of methamphetamine was reported on a mean of 18.7 (SD 9.1) days, and 62.6% also reported past 30-day use of heroin/IMF. Most (76.9%) had a history of an unintentional drug-related overdose, but 38.5% rated their current risk for an opioid overdose as none. Besides fentanyl (71.9%), toxicology analysis identified nine fentanyl analogs/metabolites (e.g., 42.7% acetyl fentanyl, 19.0% fluorofentanyl, 5.6% carfentanil), and 12.4% tested positive for Xylazine. The majority (71.4%) believed that IMF/meth was common, and 59.3% reported prior exposures to IMF/meth. 11.2% tested positive for IMF but reported no past 30-day heroin/IMF use (unknown exposure to IMF). Views that IMF/meth was common showed association with homelessness (*p* = 0.04), prior overdose (*p* = 0.028), and greater perceived risk of opioid overdose (*p* = 0.019). Self-reported exposure to IMF/meth was associated with homelessness (*p* = 0.007) and obtaining take-home naloxone (*p* = 0.025). Individuals with unknown IMF exposure (test positive for IMF, no reported past 30-day heroin/IMF use) were older (49.9 vs. 41.1 years, *p* < 0.01), and reported more frequent past 30-day use of methamphetamine (24.4 vs. 18.0 days, *p* < 0.05). They indicated lower perceived risk of opioid overdose (0.1 vs. 1.9, scale from 0 = “none” to 4 = “high,” *p* < 0.001).

**Discussion:**

This study suggests a need for targeted interventions for people who use methamphetamine and expansion of drug checking and other harm reduction services.

## Introduction

The U.S. drug-related overdose crisis continues unabated, spiking to an unprecedented 100,306 drug overdose deaths during the 12 months period ending in April 2021, almost a 30% increase compared to the year before [1]. The worsening of the overdose crisis in the U.S. is propelled by the continuing spread of illicitly manufactured fentanyl (IMF) including fentanyl analogs and other related compounds [2]. Over the past few years, overdose death data show an increasing presence of methamphetamine and other illicit stimulant positive cases, typically in conjunction with IMF, potentially signaling a new wave of the opioid overdose crisis [3, 4]. For example, from 2015 to 2019, overdose deaths involving methamphetamine have increased 180% in the U.S. [5].

Increasing methamphetamine presence in overdose mortality cases is consistent with other data sources showing growing methamphetamine use among individuals who use illicit opioids, a phenomenon that has been described as a “twin epidemic” [6–8]. On the one hand, increases in overdose mortality cases testing positive for both IMF and methamphetamine may be driven by patterns of co-use as individuals who use IMF/heroin may seek out methamphetamine for a variety of reasons and motivations, including balancing opioid effects, self-managing opioid withdrawal symptoms, and attempting to mitigate opioid-related overdose risks [4, 9, 10].

On the other hand, it has been noted that increased overdose mortality cases that test positive for methamphetamine and IMF may be due to unintentional exposures to IMF among opioid naïve individuals through IMF-contaminated (or adulterated) methamphetamine. A few prior studies have attempted to assess the scope of IMF contamination of methamphetamine supply. A study that used data from the National Forensic Laboratory Information System (NFLIS) found that the presence of IMF in the methamphetamine supply was relatively uncommon, but increased significantly between 2011 and 2016, and there were significant regional variations noted across the country ranging from 6.1% in New Hampshire, 0.9% in Ohio, and 0.2% in Virginia [11]. Between 2016 and 2019, cases with methamphetamine and IMF combinations in seized drugs increased from 239 to 1628, but they represented less than 2% of the total analyzed methamphetamine reports [12]. A study conducted in San Francisco between 2016 and 2019 with 245 women with a history of housing instability determined that the presence of fentanyl metabolites in the study population was almost entirely among women who also reported using heroin or opioid pills, and the study finding did not support the hypothesis that fentanyl was being commonly added to illicit stimulant supply in this region [13]. In contrast, qualitative interviews conducted in 2021 in Oregon with persons who used a broad range of illicit drugs noted participant views about the increasing IMF contamination of methamphetamine supply [14], but also indicated that these views were less commonly shared among individuals who were primarily using methamphetamine [15]. Overall, with continuing changes in the illicit drug supply, more data are needed to characterize experiences of individuals who use methamphetamine to help inform development of public health messaging and harm reduction interventions that could reach populations that are not routinely targeted through opioid response strategies [16].

In this study, we focus on the data we collected in Montgomery County, Ohio (City of Dayton), one of the epicenters of the opioid crisis with unintentional drug overdose death rates among the highest in the country [17]. Along with the increases in IMF-related overdose mortality in the Dayton area (Montgomery County), methamphetamine-positive unintentional drug overdose death cases also increased from 5% in 2015 to 34% in 2020, and nearly 90% of methamphetamine-positive cases have also tested positive for illicit opioids/IMFs [17]. Using structured interview and urine toxicology data from a community-recruited sample of 91 individuals who use methamphetamine, this study aims to: (a) characterize the lay views and experiences with IMF contamination of methamphetamine (IMF/meth), and (b) identify cases of unknown IMF exposures.

## Methods

Between December 2019 and November 2021, the study recruited 91 individuals who met the following criteria: (1) at least 18 years of age, (2) residing in the Dayton (OH) metro area, and (3) reporting methamphetamine use in the past 30 days. The study was approved by the Institutional Review Boards at WSU and ASU. Participants were recruited through Craigslist.org ads, referrals from other participants, flyers posted in the community, referrals from outreach workers, and through prior longitudinal study with persons with opioid use disorder [8, 18]. Participants were compensated $40 for completing an interview and $15 for referring other eligible individuals.

Interview questionnaires were developed based on preliminary qualitative research conducted with 13 persons who used methamphetamine [10]. Structured interview data were entered into RedCap (Version 9.0) [19]. The structured assessment collected sociodemographic (age, sex, ethnicity/race, education, employment, homelessness), drug use history data, drug-related overdose experiences (“How many times in your life have you experienced an unintentional drug-related overdose?”), knowledge and experiences obtaining naloxone (“Have you ever obtained Narcan/naloxone?”) and using fentanyl testing strips (“Have you ever used fentanyl testing strips?”). Perceptions of IMF/meth were assessed with the following question: “Based on your knowledge and experiences, how common are the following drug contamination issues in the Dayton area: a) methamphetamine being mixed/contaminated with fentanyl?” Response options (never; rarely; sometimes; often) were collapsed into two categories in subsequent analyses: “sometimes” or “often” responses were grouped as “common” and all other responses were grouped as “not common”. Personal exposures to IMF/meth were assessed: “How many times have you ever obtained methamphetamine that was mixed/contaminated with fentanyl?” Those who reported personal experience of obtaining methamphetamine contaminated/mixed with fentanyl, were asked: **“**How did you know you obtained or used methamphetamine that was mixed/contaminated with fentanyl?” The following response options were included, and participants were instructed to select all that apply: (1) the way it looked; (2) smell; (3) taste; (4) the way it made me feel; (5) dealer told me what it was; (6) other people told me what it was; (7) when drug tested at a treatment center or elsewhere; (8) other. Detailed information on the daily use of selected drugs (including methamphetamine, heroin, IMF) in the past 7 days was assessed to enable comparison between self-reports and urine toxicology.

All participants were asked to provide an unsupervised urine specimen to test for IMFs, methamphetamine, heroin, and other drugs. Out of 91 participants, two individuals were not able to produce a urine specimen due to self-reported dehydration associated with methamphetamine use, resulting in a sample size of 89 urine specimens. All specimen cups were labeled with a number that linked the urine specimen to the survey responses. Urine specimens were stored onsite in a refrigerator until transportation to the Montgomery County Coroner’s Office (MCCO) Toxicology laboratory. The MCCO Toxicology Laboratory is a regional laboratory that provides postmortem toxicology services to over 50 Ohio counties and is accredited by the ANSI National Accreditation Board (ANAB) in toxicology to include the toxicology specific American Board of Forensic Toxicology requirements. At the MCCO Toxicology lab, all urine specimens were analyzed using liquid-chromatography-tandem mass spectrometry (LC–MS–MS)-based methods to identify fentanyl, fentanyl analogs, metabolites, other synthetic opioids [20, 21], methamphetamine, and other drugs (complete list of drugs detected by routine testing is available: [22]). Detection methods for 24 fentanyl analogs/metabolites and subsequent application of these have been described previously [20, 21]. To identify self-report measures of heroin, fentanyl, and methamphetamine use were based on a past 3-day cut-off point for self-reported use of these drugs [23, 24].

To identify unknown exposures to IMF, we examined toxicology and self-reported data in two ways: (a) “Unknown IMF exposure, no reported past 3-day use of IMF” group included all cases that tested positive for IMF but reported no use of IMF in the past 3 days; (b) “Unknown IMF exposure, no reported past 30-day use of IMF or heroin” group included all cases that tested positive for IMF but reported no use of IMF or heroin in the past 30 days. The latter group was designed to take into account potential exposure to NPF through heroin contamination, which is common in the Dayton region [21] and to minimize recall bias.

Structured data were downloaded from Redcap, and SPSS (version 27) [25] was used to conduct statistical analyses. First, descriptive statistics (mean, standard deviation (std. dev.), frequencies) were generated to characterize the sample. Chi-square test or Fisher’s exact test for categorical variables and ANOVA or Mann–Whitney U non-parametric test for continuous variables were used to identify socio-demographic, drug use, and harm reduction service use characteristics associated with the key variables of interest: (1) views about IMF/meth (comparing individuals who thought that IMF/meth was common vs. those who did not); (2) self-reported exposure to IMF/meth; and (3) toxicology-identified cases of unknown exposures to IMF: (a) defined as cases testing positive for fentanyl or fentanyl analogs but reporting no use of IMF in the past 3 days; (b) cases tested positive for fentanyl or fentanyl analogs but reporting no use of IMF or heroin in the past 30 days.

## Results

### Sociodemographic and drug use characteristics

Out of 91 participants, 52.7% were female, 95.6% were non-Hispanic white, and the mean age was 42.4 (SD 10.6) years. The majority (73.6%) were unemployed, and 42.9% reported homelessness in the past 30 days. Average age of first methamphetamine use was 30.8 (SD 11.2). More than half (54.4%) reported injection as a primary route of methamphetamine administration in the past 30 days. Almost 63% reported using heroin/fentanyl in the past 30 days. The majority (76.9%) reported a history of an unintentional drug-related overdose, and 83.5% reported ever obtaining naloxone. Only 23.1% believed that their risk for an opioid-related overdose was high, and 38.5% believed that they were not at risk for an opioid overdose in the past 30 days (Table 1).

**Table 1:** Socio-demographics, drug use-related characteristics, as well as attitudes and experiences with illicitly manufactured fentanyl (IMF) contamination of methamphetamine (NPF/meth) (*N* = 91)

Variables	*N* or Mean	% or Standard deviation
*Sociodemographic characteristics*		
Female	48	52.7%
Age (years, mean, SD)	42.4	10.6
Non-Hispanic White	87	95.6%
Homeless (ever)	83	91.2%
*Methamphetamine (meth) and other drug use*		
Age of first meth use (mean, SD)	30.8	11.2
Days of meth use, past 30 days (mean, SD)	18.7	9.1
Meth injection (primary route of use, past 30 days)	39	54.4%
IMF and/or heroin use, past 30 days	57	62.6%
Cocaine and/or crack use, past 30 days	47	51.6%
Non-prescribed benzodiazepine use, past 30 days	36	39.6%
*Overdose (OD)*		
Ever experienced OD	70	76.9%
Perceived risk of opioid OD, past 30 days:		
None	35	38.5%
Little	13	14.3%
Moderate	13	14.3%
Somewhat High	9	9.9%
High	21	23.1%
*Use of harm reduction*		
Ever obtained take-home naloxone	76	83.5%
Ever used fentanyl testing strips	17	18.7%
*IMF contamination of methamphetamine (IMF/meth)*		
Views: IMF/meth is common^1^	65	71.4%
Ever obtained/used IMF/meth	54	59.3%
How knew that meth was contaminated with IMF^2^	N = 54	
The way it made them feel	44	81.5%
The way it looked	11	20.4%
Taste	17	31.5%
Smell	6	11.1%
Drug testing (e.g., urine testing by a treatment provider)	16	29.6%
Dealer told me	4	7.4%
Other people told me	11	20.4%
Other	3	6.8%
*Toxicology-identified cases of unknown IMF exposures*		
Unknown IMF exposure, no reported past 3-day use of IMF	16	18.0%
Unknown IMF exposure, no reported past 30-day use of IMF	10	11.2%

^1^“Common” included response options “sometimes” or “often.”

^2^Participants could select more than one response options (all that apply)

### Attitudes and experiences with IMF/meth

Out of all 91 participants, 71.4% shared views that methamphetamine contamination with IMF was common (rated as occurring “sometimes” or “often”), and 59.3% indicated that they had personal exposures to IMF/meth (Table 1). Most commonly, participants indicated knowing that they were exposed to IMF/meth from the way it made them feel (70.4% out of 54 individuals who reported such experiences) and close to 30% indicated that they knew about potential contamination after undergoing drug testing at a treatment facility or elsewhere (Table 1).

Table 2 presents bivariate associations between selected sociodemographic and drug use characteristics and (1) attitudes that IMF/meth was common; (2) self-reported exposure to IMF/meth. Greater proportion of participants who viewed IMF/meth as common had lifetime experiences of homelessness (95.4% vs. 80.8%, *p* = 0.04) and drug-related overdose (83.1% vs. 61.5%, *p* = 0.028). They also rated their overdose risks greater than those who did not view IMF/meth as common (1.9 vs. 1.0, on a scale from 0- no risk to 4-high risk, *p* = 0.019). Individuals who viewed IMF/meth as common were also significantly more likely to self-report exposure to IMF/meth (70.8% vs. 30.8%, *p* < 0.001). Self-reported exposure to IMF/meth was linked to greater proportion of participants reporting a history of homelessness (98.1% vs. 81.1%, *p* = 0.007), prior experiences of obtaining take-home naloxone (90.7% vs. 73.0%, *p* = 0.025), as well as views that methamphetamine contamination with IMF was common (85.2% vs. 51.4%, *p* < 0.001).

**Table 2:** Socio-demographic and drug use characteristics associated with attitudes and self-reported exposures to IMF-contaminated methamphetamine (IMF/meth) in a community-recruited sample of persons who use methamphetamine (*N* = 91)

Variables	Viewed NPF/meth as common n (%) or mean (SD)			Self-reported exposure to NPF/meth n (%) or mean (SD)		
	Yes *N* = 65	No *N* = 26	ANOVA or Chi-Squared, p value	Yes *N* = 54	No *N* = 37	ANOVA or Chi-Squared, *p* value
Age	42.14 (11.1)	43.00 (9.7)	0.439^^^	42.7 (9.4)	42.0 (12.3)	0.747
Females	38 (58.5%)	10 (38.5%)	0.840	30 (55.6%)	18 (48.6%)	0.517
Ever homeless	62 (95.4%)	21 (80.8%)	**0.040**^**#**^	53 (98.1%)	30 (81.1%)	**0.007**^**#**^
Unemployed	49 (75.4%)	18 (69.2%)	0.547	43 (79.6%)	24 (64.9%)	0.116
Days of meth use, past 30 days	19.0 (9.2)	17.8 (9.1)	0.573^^^	19.8 (9.4)	17.0 (8.6)	0.174^^^
Injection as primary admin of meth, past 30 days	29 (44.6%)	10 (38.5%)	0.592	25 (46.3%)	14 (37.8%)	0.423
Days of heroin use, past 30 days	10.9 (12.8)	6.8 (11.4)	0.285^^^	8.5 (11.3)	11.5 (14.0)	0.576^^^
Days of IMF use, past 30 days	13.4 (13.1)	10.1 (13.4)	0.224^^^	11.5 (12.5)	13.8 (14.3)	0.703^^^
Days of powder cocaine use, past 30 days	2.2 (4.9)	3.6 (8.3)	0.900^^^	2.6 (6.2)	2.6 (6.0)	0.733^^^
Days of crack cocaine use, past 30 days	2.5 (5.8)	4.3 (8.6)	0.739^^^	3.2 (6.9)	2.8 (6.6)	0.244^^^
Ever experienced an overdose	54 (83.1%)	16 (61.5%)	**0.028**	41 (75.9%)	29 (78.4%)	0.785
Perceived risk of opioid overdose (0-none to 4-high), past 30 days	1.9 (1.6)	1.0 (1.5)	**0.019**^**^**^	1.8 (1.7)	1.4 (1.5)	0.305^^^
Ever obtained take-home naloxone	57 (87.7%)	19 (73.1%)	0.090	49 (90.7%)	27 (73.0%)	**0.025**
Ever used fentanyl testing strips	13 (20.0%)	4 (15.4%)	0.769^^^	9 (16.7%)	8 (21.6%)	0.551
Views: Meth contamination with IMF is common (sometimes/often)				46 (85.2%)	19 (51.4%)	** < 0.001**
Ever obtained/used meth contaminated with IMF	46 (70.8%)	8 (30.8%)	** < 0.001**			

^^^Mann–Whitney U non-parametric test

^#^Fisher’s Exact Test

Bold text indicates statistically significant values (*p*≤0.05)

### Results of urine toxicology and unknown exposure to IMF

Results of urine toxicology analysis are presented in Table 3. Out of 89 participants, 71.9% tested positive for fentanyl. In addition to fentanyl, toxicology analysis identified ten fentanyl analogs/metabolites in urine samples of the study participants, including acetylfentanyl (42.7%), fluorofentanyl (19.0%), carfentanil (5.6%), and others (Table 3). 12.4% tested positive for Xylazine. Past 3-day use of methamphetamine was reported by 82.4%, of the sample, and 87.6% were identified as methamphetamine positive through urine toxicology analysis. Past 3-day use of IMF was reported by 52.7% of the sample, but nearly 72% showed positive IMF urine toxicology results.

**Table 3:** Results of urine toxicology analysis and comparison to self-reported use of IMF, heroin, methamphetamine, and other drugs (N = 89)

Drugs/metabolites	All participants *N* = 89		Unknow IMF exposure, no reported past 3-day IMF use^1^ *N* = 16		Unknown IMF exposure, no reported past 30-day heroin/IMF use^2^ *N* = 10	
	Results of urine toxicology analysis	Self-reported use in the past 3 days	Results of urine toxicology analysis	Self-reported use in the past 3 days	Results of urine toxicology analysis	Self-reported use in the past 3 days
Methamphetamine (and/or amphetamine)	78 (87.6%)	73 (82.0%)	16 (100.0%)	16 (100.0%)	10 (100.0%)	10 (100.0%)
Cocaine	37 (41.6%)	24 (27.0%)	9 (56.3%)	3 (18.8%)	4 (40.0%)	1 (10.0%)
Benzodiazepines	16 (18.0%)	20 (22.5%)	5 (31.3%)	3 (18.8%)	4 (40.0%)	2 (20.0%)
Heroin (morphine/6-MAM)	17 (19.1%)	26 (29.2%)	2 (12.5%)	3 (18.8%)	1 (10.0%)	0 (0%)
IMF (fentanyl and/or analogs, any):	64 (71.9%)	48 (53.9%)	16 (100.0%)	0 (0.0%)	10 (100.0%)	0 (0%)
Fentanyl (and/or norfentanyl metabolite)	64 (71.9%)	48 (53.9%)	16 (100.0%)	0 (0.0%)	10 (100.0%)	0 (0%)
Carfentanil	5 (5.6%)	6 (6.7%)	1 (6.3%)	0 (0.0%)	0 (0.0%)	0 (0%)
Despropionylfentanyl (4-ANNP)	51 (57.3%)		10 (62.5%)		7 (7.0%)	
Acetylfentanyl	38 (42.7%)		7 (43.8%)		3 (30.0%)	
Fluorofentanyl	17 (19.1%)		5 (31.3%)		4 (40.0%)	
Despropionyl Fluorofentanyl	16 (18.0%)		6 (37.5%)		4 (40.0%)	
Tetrahydrofuran Fentanyl	12 (13.5%)		0 (0.0%)		0 (0.0%)	
Benzylfentanyl	10 (11.2%)		1 (6.3%)		1 (10.0%)	
Valeryl/Isovaleryl Fentanyl	6 (6.7%)		0 (0.0%)		0 (0.0%)	
Butyryl/Isobutyryl Fentanyl	2 (2.2%)		0 (0.0%)		0 (0.0%)	
Acrylfentanyl	1 (1.1%)		0 (0.0%)		0 (0.0%)	
**X**ylazine	11 (12.4%)		2 (12.5%)		1 (10.0%)	

^1^Test positive for IMF but reported no use of IMF in the past 3 days

^2^Test positive for IMF but reported no use of heroin or IMF in the past 30 days

Overall, 18.0% (16 out of 89 individuals) were identified as unknown IMF exposure cases, with no reported past 3-day use of IMF. All 16 cases reported past 3-day use of methamphetamine. However, it is not possible to know with certainty the source of potential NPF contamination for all 16 individuals since they reported complex patterns of polydrug use, including heroin (*n* = 3) and cocaine (*n* = 3) use in the past 3 days (Table 3).

Ten individuals (11.2%) were identified as unknown IMF exposure cases with no reported past 30-day use of IMF or heroin. All 10 tested positive methamphetamine, but other drug use was also self-reported and/or identified through urine toxicology, including 4 individuals testing positive for cocaine (Table 3).

Toxicology-identified cases of unknown IMF exposure with no reported past 3-day use of IMF were more likely to be older (48.1 vs. 40.7 years of age, *p* < 0.001). They reported less frequent past 30-day use of heroin (4.1 vs. 11.2 days, *p* = 0.040), IMF (0.9 vs. 15.3 days, *p* < 0.001), and cocaine (0.3 vs. 3.2 days, *p* = 0.035). Individuals with unknown IMF exposure reported significantly lower perceived risk of opioid overdose (mean of 0.8 vs. 1.9 on a scale from 0- no risk to 4 high risk, *p* < 0.011). (Table 4).

**Table 4:** Socio-demographic and drug use characteristics associated toxicology-identified cases of unknown IMF exposure in a community-recruited sample of persons who use methamphetamine (N = 89)

Variables	Test positive for IMF but report no use of IMF in the past 3 days n (%) or mean (SD)			Test positive for IMF but report no use of IMF or heroin in the past 30 days n (%) or mean (SD)		
	Yes *N* = 16	No *N* = 73	ANOVA or Chi-Squared, *p* value	Yes *N* = 10	No *N* = 79	ANOVA or Chi-Squared, *p* value
Age	48.1 (6.5)	40.7 (10.8)	**0.001^**	49.9 (6.9))	41.1 (10.5)	**0.003^**
Females	11 (68.8%)	36 (49.3%)	0.158	7 (70%)	40 (50.6%)	0.323^#^
Ever homeless	15 (93.8%)	68 (93.2%)	1.000^#^	9 (90.0%)	74 (93.7%)	0.522^#^
Unemployed	10 (62.5%)	55 (75.3%)	0.354	5 (50.0%)	60 (75.9%)	0.081
Days of meth use, past 30 days	21.9 (8.1)	18.0 (9.2)	0.098^	24.4 (7.7)	18.0 (9.0)	**0.030^**
Injection as primary admin of meth, past 30 days	7 (43.8%)	32 (43.8%)	0.995	3 (30.0%)	36 (45.6%)	0.503^#^
Days of heroin use, past 30 days	4.1 (8.7)	11.2 (12.9)	**0.040^**	0.0 (0.0)	11.2 (12.8)	
Days of IMF use, past 30 days	0.9 (2.4)	15.3 (13.3)	** < 0.001^**	0.0 (0.0)	14.3 (13.2)	
Days of powder cocaine use, past 30 days	0.3 (0.8)	3.2 (6.6)	**0.035^**	0.1 (0.3)	3.0 (6.4)	**0.034^**
Days of crack cocaine use, past 30 days	1.2 (3.3)	3.5 (7.3)	0.067^	0.7 (1.9)	3.4 (7.1)	0.151^
Ever experienced an overdose	10 (62.5%)	58 (79.5%)	0.148	8 (80.0%)	60 (75.9%)	1.000^#^
Perceived risk of opioid overdose (0-none to 4-high), past 30 days	0.8 (1.4)	1.9 (1.6)	**0.011**^**^**^	0.1 (0.3)	1.9 (1.6)	** < 0.001^**
Ever obtained take-home naloxone	12 (75.0%)	63 (86.3%)	0.269^#^	8 (80.0%)	67 (84.8%)	0.654^#^
Ever used fentanyl testing strips	4 (25.0%)	13 (17.8%)	0.497^#^	3 (30.0%)	14 (17.7%)	0.395^#^
Views: Meth contamination with IMF is common	10 (62.5%)	55 (75.3%)	0.294	7 (70.0%)	58 (73.4%)	1.000^#^
Ever obtained/used meth contaminated with IMF	10 (62.5%)	44 (60.3%)	0.869	6 (60.0%)	48 (60.8%)	1.000^#^

^^^Mann–Whitney U non-parametric test

^#^Fisher’s exact test

Bold text indicates statistically significant values (*p*≤0.05)

Similarly, toxicology-identified cases of unknown IMF exposure with no reported past 30-day use of heroin or fentanyl were also more likely to be older (49.9 vs. 41.1 years, *p* < 0.01), reported more frequent past 30-day use of methamphetamine (24.4 vs. 18.0 days, *p* < 0.05), but less frequent use of cocaine (0.1 vs. 3.0 days, *p* < 0.05). They indicated lower perceived risk of opioid overdose (0.1 vs. 1.9, scale from 0 = ”none” to 4 = ”high,” *p* < 0.001) (Table 4).

## Discussion

Our study findings demonstrate that IMF/meth is viewed and experienced as a common occurrence in the local illicit drug market, with about 60% of the sample indicating that they experienced personal exposures to IMF/meth. IMF contamination of methamphetamine supply presents significant risks, especially for individuals who are opioid-naïve or have lower tolerance for opioid use. Additionally, IMF/meth also presents significant risks to persons who use heroin/IMF because such contamination scenarios make IMF and other drug dosing more unpredictable, and difficult to “calibrate” and adjust. Exposures to IMF/meth are especially troubling in the context of lay beliefs among some individuals that use of methamphetamine could help prevent or reverse IMF-related overdose [9, 26].

About 30% of participants indicated that they knew about potential exposure to IMF/meth from the drug testing results at a treatment facility or elsewhere. Given high unpredictability of drug supply, it is imperative for treatment and other programs that rely on urine drug testing for identification of ongoing substance use to communicate and discuss testing results back to their clients to help inform about potential drug contamination issues and risks.

The study is one of the first to explore lay experiences with IMF and methamphetamine use in the Midwestern U.S. and to conduct toxicological analyses to test for a broad range of fentanyl analogs in a community-recruited sample of individuals who use methamphetamine. Our data also demonstrate that the study participants were exposed to a large variability of IMF-type drugs. Besides fentanyl and despropionylfentanyl (4-ANPP -precursor chemical/metabolite of IMF), there were nine types of fentanyl analogs identified in the urine samples of the study participants, including fluorofentanyl (19.0%) and carfentanil (5.6%). In our prior study with individuals who use heroin/fentanyl conducted in 2017–2018, similar variability in IMF-related drugs was identified, although different types of fentanyl analogs showed greater prevalence; for example, 47.5% tested positive for carfentanil and there were no fluorofentanyl-positive cases [21]. Over 12% also tested positive Xylazine, a nonopioid veterinary anesthetic and sedative that may pose increased risks of overdose and other health harms to individuals who use opioids and other drugs [27]. Xylazine is increasingly detected in illicit opioid and other drug supply in the US [28], and there is an urgent need for improved testing capacity in forensic, medical, and harm reduction settings (e.g., Xylazine test strips) to detect Xylazine exposures and develop harm mitigation strategies to address the risks.

Among 91 study participants, 18% were toxicology-identified cases of unknown exposure to IMF (no reported past 3-day use of IMF). About 11% tested positive for IMF but reported no use of heroin or IMF in the past 30 days. These numbers are notably greater compared to a prior study conducted in San Francisco that found only one fentanyl positive case (0.3%) among individuals who reported methamphetamine or cocaine use but did not use illicit opioid [13]. Individuals with unknown IMF exposure were older, possibly indicating generational differences in drug knowledge and preferences with older individuals being less connected to the social networks and sources of information on the emerging trends of IMF use and “homegrown” strategies to recognize IMF contamination or adulteration cases. Prior studies have consistently shown that older age was related to decreased preference for IMF-containing drugs [29, 30]. These findings suggest the need for harm reduction interventions that recognize and address unique vulnerabilities and service delivery needs that are tied in with generational and age-related differences.

Overall, participants reported complex patterns of polydrug use, and it is difficult to attribute the source of IMF contamination to methamphetamine and/or other drugs. However, individuals with unknown IMF exposure tended to report less frequent use of heroin, IMF, and cocaine. Individuals who tested positive for IMF but reported no use of IMF or heroin in the past 30 days also reported more frequent use of methamphetamine. Importantly, our findings indicate that individuals with toxicology-identified unknown IMF exposure were significantly less likely to perceive themselves at risk for opioid-related overdose. A study conducted in Oregon also noted that individuals who were primarily using methamphetamine viewed their risk of opioid overdose as low [15]. These findings indicate the need for increased attention to opioid-related overdose education interventions, including community naloxone distribution, that target individuals who use methamphetamine and other non-opioid drugs such as cocaine.

People with a history of homelessness were more likely to view IMF/meth as common and they were also more likely to self-report personal exposures to IMF/meth. Homelessness increases vulnerability to a range of health and social harms, including increased unpredictability of drug access and greater difficulty of navigating local risk environments [31]. This finding makes a further case for the importance of harm reduction support services, such as naloxone and fentanyl test strips, in shelters or support centers for unhoused people [32]. On the other hand, it also highlights the need for increased focus on social and economic factors that contribute to the root causes of drug use and development of drug use disorders [33].

Prior experiences of overdose were associated with the attitudes that IMF/meth was common. It is not clear if reported overdose experiences were due to cases of IMF and methamphetamine contamination. However, a history of overdose may signal more risky drug use environments and patterns of use that may lead to greater likelihood of exposures to less reliable drug supply. Individuals who had prior experiences of obtaining take-home naloxone were also more likely to report personal exposures to IMF/meth. This association may indicate that individuals with personal adverse experiences of obtaining contaminated drugs are potentially more likely to reach out for access to harm reduction measures such as take-home naloxone kits. Harm reduction efforts targeted at wider community naloxone distribution are needed, as overdose risk has expanded beyond individuals who primarily use opioids.

Overall, the sample had low prevalence of fentanyl testing strip (FTS) use (15%) which may reflect overall low levels of provision of FTS by the local harm reduction organizations at the time of data collection, including prior to the COVID-19 pandemic [34]. FTS distribution was also limited because of the state laws that classify FTS as drug paraphernalia [35, 36]. Along with policy changes to decriminalize FTS, more research is needed to help increase FTS utilization among individuals who use illicit stimulants [35]. On the other hand, our findings also highlight the limitations of FTS in the context of drug supply that is saturated with a wide range of fentanyl analogs, as seen in our sample. FTS provide qualitative identification of fentanyl and some analogs but cannot differentiate between different types of analogs [37]. Because fentanyl analogs vary significantly in their potency, information on specific analog detection is crucially important to help individuals assess and navigate overdose-related risks. For example, while acetyl fentanyl has lower potency than fentanyl, carfentanil is estimated to have 100 times greater potency compared to fentanyl [38]. In addition, FTS use for testing methamphetamine samples requires specific instructions on increased sample dilution to avoid false positives [39]. Our findings indicate the need for improved FTS-type tools for drug checking that can be reliable and specific in identifying emerging fentanyl analogs and other novel synthetic opioids. In addition, there is an urgent need to address policy and other implementation barriers for expansion of advanced community-based drug checking and surveillance services that rely on portable spectrometry devises to provide point-of-service analysis of the content of illicit drugs [36].

Limitations of this study include recall bias when reporting drug use in the past 3 days for comparison to toxicology findings. In addition, individual metabolic and drug use factors may contribute to variations in detection window for selected drugs by urine toxicology [40]. For example, prior research conducted in a hospital settings has shown that at 48-h post-fentanyl administration, fentanyl metabolites were detectable in all studied patients, and at 96-h, about half still tested positive for fentanyl metabolites [41]. Furthermore, we recognize our limitations in tracing back the source of IMF contamination since participants reported complex patterns of polydrug use. Additionally, our sample was relatively small and not randomly recruited. Research with larger samples is needed to track lay experiences and behaviors related to methamphetamine use. In addition, our sample included mostly non-Hispanic White individuals. Although the sample largely resembles demographic profiles of people who use methamphetamine identified by other epidemiological studies in Ohio [8, 17], more research is needed to understanding IMF contamination of stimulants in the context of race/ethnicity and paying attention to distinct regional contexts along with differentiation by stimulant types (e.g., methamphetamine, powdered cocaine, crack cocaine) [42].

Our research findings highlight high unpredictability of illicit methamphetamine and other drug supply in the Dayton, Ohio region. It is not clear why IMF may be added to methamphetamine. It is possible that some of these cases may be due to unintentional cross contamination due handling multiple drugs [43]. As previously suggested [35], more in-depth qualitative research is needed with persons involved in distribution of illicit drugs to identify reasons for IMF contamination of methamphetamine and other non-opioid drugs. Along with the continuing expansion of access to naloxone, it is crucial for the community overdose prevention programs to develop outreach and intervention approaches to reach individuals who use methamphetamine and other non-opioid drugs. These strategies need to build on community-based knowledge and grassroots techniques for navigating IMF-related risks [26]. Our findings emphasize the need for policy changes to facilitate the expansion of novel harm reduction interventions, including community-based drug checking services [36] and overdose prevention centers [44], which are safe places where people can come to use previously purchased drugs in a controlled environment and with available links to health and social services [45]. Such programs have been implemented in Canada, Australia, and in some European countries, but they still face significant legal challenges in the U.S. [46]. Importantly, our findings also emphasize the need for overdose prevention centers to address complex patterns of opioid, stimulant and other drug use and provide space for different modes of drug consumption, including smoking, injection, and inhalation [44]. Continued research and monitoring is needed to characterize shifting trends and risks associated with unpredictability of methamphetamine and other drug supply in the context of growing proliferation of IMF and related compounds.
